# Displaced center of pressure on the treated side in individuals with essential tremor after radiofrequency ablation: a longitudinal case–control study

**DOI:** 10.3389/fneur.2023.1182082

**Published:** 2023-06-29

**Authors:** Atsuya Sato, Takaomi Taira, Kazuya Kitada, Toshiki Ando, Toyohiro Hamaguchi, Michiko Konno, Yoshinori Kitabatake, Toshiyuki Ishioka

**Affiliations:** ^1^Department of Occupational Therapy, School of Rehabilitation, Tokyo Professional University of Health Sciences, Tokyo, Japan; ^2^Graduate School of Health and Social Services, Saitama Prefectural University, Saitama, Japan; ^3^Department of Rehabilitation, Sanai Hospital, Saitama, Japan; ^4^Department of Neurosurgery, Tokyo Women's Medical University, Tokyo, Japan

**Keywords:** center-of-pressure, essential tremor, post-operative symptoms, radiofrequency ablation, ventral intermediate thalamic nucleus

## Abstract

**Background:**

Essential tremor (ET) is a common involuntary movement disorder (IMD). Radiofrequency ablation (RFA) targeting the ventral intermediate nucleus (Vim) of the thalamus is a stereotactic neurosurgery performed in individuals with ET when pharmacotherapy is no longer effective. Though the reasons remain largely unclear, certain adverse events are known to appear post-RFA. These may be due to functional changes in the Vim, related to RFA-induced tremor reduction, or an adverse reaction to compensatory movement patterns used to perform movements in the presence of tremor symptoms.

**Objective:**

This study aimed to understand the characteristics of post-RFA symptoms in individuals with ET.

**Methods:**

In a longitudinal case–control study, we compared post-RFA symptoms between individuals with ET who underwent Vim-targeted RFA and those with IMD who underwent non-Vim-targeted RFA. Symptoms were compared preoperatively and 1-week and 1-month postoperatively. Quantitative assessments included center-of-pressure (COP) parameters, grip strength, Mini-Mental State Examination, two verbal fluency tests, and three types of physical performance assessments (upper extremity ability, balance ability, and gait ability).

**Results:**

Individuals with ET after RFA showed horizontal displacements of the COP to the treated side (the dominant side of the RFA target's hemisphere) at 1-week postoperatively compared to the preoperative period. The horizontal COP displacement was associated with balance dysfunction related to postural stability post-RFA. Other COP parameters did not significantly differ between the ET and IMD groups.

**Conclusion:**

COP displacement to the treated side may be due to a time lag in adjusting postural holding strategies to the long-standing lateral difference in tremor symptoms associated with tremor improvement after RFA.

## 1. Introduction

Essential tremor (ET) is a common neurological disorder, affecting people of all ages, with a worldwide prevalence of 4.6% in people aged ≥65 years as reported in a meta-analysis (1). It is characterized by postural and motion tremors (4–12 Hz), involving the hands, forearms, and head (2) often affecting activities of daily living. ET has recently been recognized as a monosymptomatic disorder; however, it is also associated with other symptoms, such as gait disturbance, balance impairment, and non-motor symptoms (3–5).

Treatment for ET generally begins with lifestyle guidance, including stress management. Patients with inadequate response are switched to pharmacotherapy, particularly when symptoms are sufficiently severe to interfere with activities of daily living (ADLs) and social participation. Rehabilitation for tremors has been reported as an intervention for movement disorders (6), which includes a selection of adaptive self-help devices for tremors and training to use them (7). In patients who fail to improve with conventional pharmacologic therapy, chemo-denervation with botulinum toxin, and stereotactic neurosurgery may significantly ameliorate ET. There are two common types of stereotactic neurosurgeries: stimulation and coagulation. Deep brain stimulation (DBS) is a technique, in which a stimulating electrode is implanted in the targeted brain nucleus. Radiofrequency ablation (RFA) is a coagulative technique (8), involving stereotactic insertion of electrodes into the ventral intermediate thalamic nucleus (Vim), the target site, to create RFA foci to improve tremor symptoms.

DBS is currently the mainstay of treatment because of the possibility of postoperative stimulation adjustments for symptoms and complications (9). However, certain patients develop bacterial infections with the implantation of the device or do not respond to DBS stimulation. In those cases, RFA, which shows a long-time effect, is handy as a salvageable option as an alternative to DBS. Swelling surrounding the permanent brain lesion during the RFA process was reported to be a transient neurological deficit related to the neural network involved in the Vim, leading to ataxia, hemiparesis, dysarthria, and cognitive decline (10–19), which may require postoperative rehabilitation other than those for tremors. Although it makes sense to advance our understanding of post-RFA symptoms, only the appearance of symptoms has been investigated and not fully verified. Understanding the characteristics of post-RFA symptoms can help to clarify priorities for intervention during postoperative rehabilitation and to identify signs that require intensive intervention.

This study, therefore, investigated the characteristics of symptoms occurring after RFA for ET by quantitatively appropriate endpoints. To explain postoperative symptoms, we compared perioperative symptoms among ET individuals who underwent Vim-targeted RFA and individuals with involuntary movement disorders (IMDs) who underwent RFA targeting other regions.

## 2. Materials and methods

### 2.1. Study design

This was a single-center, longitudinal case–control study. It was approved by the Ethical Review Committee of the Tokyo University of Health Sciences (TPU-20-004), the institution where the research was conducted, and by the Sanai Hospital Ethical Review Committee (21-s001), which was the hospital responsible for conducting the surgeries and collecting data before and after surgery. All participants provided written informed consent after being given a complete description of the study.

### 2.2. Sample size

Since we were unable to find any previous report that quantitatively evaluated the complication symptoms and motor and non-motor functions before and after surgery in individuals with ET or IMD, we calculated the sample size using G Power 3.1 (https://www.psychologie.hhu.de/arbeitsgruppen/allgemeine-psychologie-und-arbeitspsychologie/gpower), with the following assumptions: effect size (f) = 0.25, significance level (p) = 0.05, power = 0.8, and measurement time-points = 3. The calculated minimal number of participants in each group was 14 (28/2). We set the number of participants to 15 per group, taking dropouts into account.

### 2.3. Participants

The participants were inpatients who underwent RFA at Sanai Hospital in Saitama City between February and December 2020. The inclusion criteria were individuals with ET, among whom Vim was the targeted RFA site (ET group, 15 patients), and those with other IMDs, such as dystonia, among whom non-Vim regions, such as the ventral oral nucleus (Vo), globus pallidus internus (GPi), and pallido-thalamic tract (PTT), were the RFA targets (IMD group, 15 patients). Exclusion criteria were apparent cognitive impairment (Mini-Mental State Examination [MMSE] scores ≤ 24) and previous stereotactic brain surgery.

One neurosurgeon (T.T.) performed all surgeries and prescribed rehabilitation.

### 2.4. Evaluations

Evaluations were performed on the day before surgery (pre-op) within 1 week postoperatively (1wpost-op) and approximately 1 month postoperatively (1mpost-op). One physical therapist (K.K.) and one occupational therapist (T.A.) performed all evaluations. The method of instruction during the evaluations was manualized and implemented so that there would be no discrepancies between the examiners.

#### 2.4.1. Tremor function, ADL, and quality of life in ET individuals

The Essential Tremor Rating Assessment Scale (TETRAS) comprises a 9-item performance subscale (TETRAS-PS) and a 12-item ADL subscale (TETRAS-ADL) (20). The Quality of Life in Essential Tremor Questionnaire (QUEST) is a self-reporting questionnaire consisting of five domains (communication, work and finances, hobbies and leisure, physical, and psychosocial) comprising 30 items (21). The higher the score, the more severe the tremor symptoms and the lower the ADL ability and quality of life (QOL). These scales were used to assess individuals in the ET group only.

#### 2.4.2. Motor symptoms

**Figure 1:**
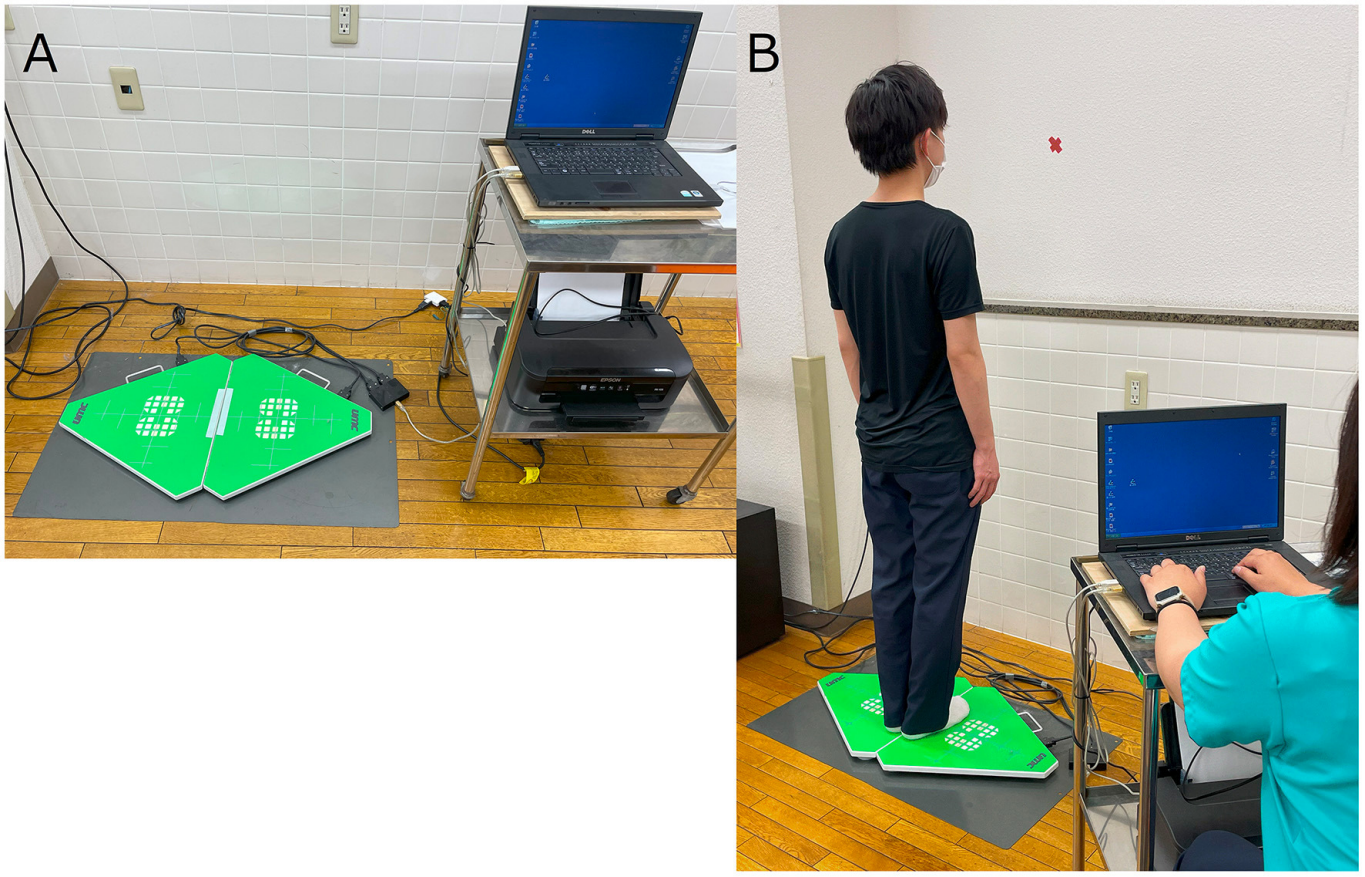
Methods for the measurement of the center of pressure (COP). **(A)** Force platform used in the study. **(B)** Arrangement for COP measurement.

The quantitative postural assessment was performed using a force platform (UM-BAR II, UNIMEC, Usmate Velate, Italy) that measures the center of pressure (COP), which corresponds to the center of gravity response (Figure 1A). The COP using the platform was selected based on previous studies (22–24). We measured the root mean square area, the total path length of COP (COP-RMA and COP-LNG, respectively), and the displacement of the COP in the X- and Y-axis directions (COP-X and COP-Y, respectively). Participants were instructed to stand on the force platform with their feet together, eyes open, and look at the gazing point (marked with a red X) on the wall in front of them (Figure 1B). For comparison with the COP displacement to the treated side (for instance, the dominant limb side of the cortical hemisphere operated on as the RFA target), positive values were assigned to the displacement on the treated side.

Grip strength (muscle strength) was determined using a hand dynamometer as the average of three measurements on the treated side (dominant limb of RFA target's cortical hemisphere) and non-treated side (same limb side of RFA target's cortical hemisphere).

#### 2.4.3. Non-motor symptoms

As a non-motor symptom, cognitive dysfunction was assessed using the MMSE and two types of verbal fluency tests: category-cued (VF-c) and phonologically cued (VF-p) tests. We recorded the MMSE score, the number of words expressed per minute on the VF-c (listing vegetable names) and on the VF-p (listing words beginning with “ka”).

#### 2.4.4. Physical performance

Gait ability was measured using the 10-meter walk test (10-MWT). The time required by participants to walk 10 m as fast as possible was measured (25). The walking distance before the measurement was 3 m, used in Japanese clinical practice, instead of the original 5 m (25). Balance ability was tested using 14 tasks in the Berg Balance Scale (BBS) (26). Upper extremity performance was evaluated using the Simple Test of Upper Extremity Function (STEF), a Japanese standardized test of 10 items scored from 0 to 10 for each hand (total score: 0–100). We obtained STEF scores for both sides (27). A higher score indicated better upper limb manipulation ability.

### 2.5. Statistical methods

We compared the preoperative age, disease duration, and age of onset in the ET and IMD groups using Student's *t*-test. We compared sex distribution using Fisher's exact test. Multiple imputations (the Markov chain Monte Carlo [MCMC] method) were used to address missing data, assuming that data were missing at random. To ensure internal validity, five datasets with missing values were created and analyzed using SPSS (ver. 27.0; IBM Inc., Armonk, NY, USA). The average values obtained in these datasets were assigned as the missing values for the analyses.

We conducted a linear mixed model analysis to detect improvement in tremor symptoms after RFA, using the TETRAS-PS, TETRAS-ADL, and QUEST scores as dependent variables and the measurement time-point as the independent variable (pre-op, 1wpost-op, and 1mpost-op), including age and sex as covariates and participants as random effects. After confirming that the created model was statistically more significant than the null model, a test was performed to reveal substantial differences in the dependent variables according to the measurement time-point.

To analyze changes in motor symptoms (COP and grip strength), non-motor symptoms (MMSE, VF-c, and VF-p), and physical performance (10MWT, BBS, and STEF) after RFA, we also evaluated interactions between measurement time points and group and also the main effects of each measurement time-point and group. A linear mixed model was used to examine changes from before to after RFA, including age and sex as covariates (with weight added as a covariate for grip strength), and participants as random effects. After confirming that the created model was statistically more significant than the null model, a test was performed to detect significant differences in the interaction and main effect of group (ET vs. IMD) and measurement time-point (pre-op, 1wpost-op, and 1mpost-op) on the objective variable.

To demonstrate the relationship between physical performance decline and the items of motor or non-motor symptoms that showed interaction in the linear mixed model, we analyzed correlations using the amount of change in the measurements from the preoperative level in each group.

Unless otherwise stated, statistical analyses were performed using Jamovi ver. 1.6.23 (https://www.jamovi.org/download.html). Statistical significance was defined as a *p*-value of < 0.05.

## 3. Results

The mean age and disease duration among the groups were significantly different: the mean age was higher, and the duration of the disease was longer in the ET group than in the IMD group (Table 1).

**Table 1:** Demographic and clinical characteristics of participants with ET and IMD.

	**ET (*n =* 15)**	**IMD (*n =* 15)**	***P-*value**
Age, Mean (SD) years	56.5 (18.2)	43.7 (15.3)	**0.046** ^**a**^
Female, *n* (ratio %)	6 (40.0)	11 (73.3)	0.139^b^
Diagnosis, *n*			
(Essential tremor/cervical dystonia/writer's cramp/trunk dystonia)	15/0/0/0	0/4/9/2	—
Onset age, mean (SD) years	34.7 (21.9)	36.2 (14.4)	0.830^a^
Duration of disease, mean (SD) years	24.3 (15.1)	7.4 (9.7)	**0.001** ^**a**^
Target site, *n* (right/left)	4/11	3/12	>0.999^b^
Target, *n* (Vim/Vo/PTT/GPi)	15/0/0/0	0/9/3/3	—

ET, Essential tremor with Vim-targeted radiofrequency ablation; GPi, globus pallidus internus; IMD, Involuntary movement disorders with non-Vim-targeted radiofrequency ablation; PTT, pallidothalamic tract; SD, Standard deviation; Vim, ventral intermediate thalamic nucleus; Vo, ventral oral. ^a^*t*-test, ^b^Fisher's exact test, —: No data. The number of individuals with ET in terms of onset age and duration of disease was 14 because the onset age of one participant was unknown. Bold font indicates significant correlations.

### 3.1. Evaluations

Complete data were available for 24 participants. For six participants, some data were missing. One participant had no data at 1wpost-op, and two participants had no data at 1mpost-op. Three participants had no data on non-motor symptoms at any time point, and the other had no data on 10-MWT at 1mpost-op. We used the MCMC method to impute missing values and then conducted linear mixed models.

### 3.2. Tremor function, ADL, and QOL in ET individuals

A main effect of measurement time-point in the tremor assessment was observed for all three endpoints in the ET group (TETRAS-PS: F = 39.76, *p* < 0.001, TETRAS-ADL: F = 35.70, *p* < 0.001, and QUEST: F = 17.955, *p* < 0.001). Performance in both the TETRAS and QUEST had improved at 1wpost-op and 1mpost-op than that pre-op (*t*-value range: 4.40–7.918, all *p*-values *p* < 0.001), as shown in Table 2.

**Table 2:** Tremor function, ADL, and QOL in participants with essential tremor post-radiofrequency ablation.

**Variables above: mean (SE) below: estimated marginal mean (SE)**	**Pre-op**	**1-week post-op**	**1-month post-op**	**Main effect of measurement time-point**	***Post-hoc***
TETRAS-PS	21.3 (2.02) 20.75 (1.84)	5.86 (1.87) 5.34 (1.84)	5.03 (1.84) 4.51 (1.84)	**F**_**(2, 28)**_ **=** **39.76** ***p** **<*** **0.001**	**pre-op vs. 1wpost-op (t** **=** **7.512**, ***p** **<*** **0.001) pre-op vs. 1mpost-op (t** **=** **7.918**, ***p** **<*** **0.001)**
TETRAS-ADL	18.9 (1.93) 18.43 (1.75)	7.51 (1.92) 7.07 (1.75)	6.62 (1.69) 6.19 (1.75)	**F**_**(2, 28)**_ **=** **35.70**, ***p** **<*** **0.001**	**Pre-op vs. 1wpost-op (t** **=** **7.030**, ***p** **<*** **0.001) pre-op vs. 1mpost-op (t** **=** **7.576**, ***p** **<*** **0.001)**
QUEST	33.1 (3.86) 32.5 (4.09)	17.9 (4.40) 17.3 (4.09)	13.4 (3.88) 12.7 (4.09	**F**_**(2, 28)**_ **=** **17.955** ***p** **<*** **0.001**	**pre-op vs. 1wpost-op (t** **=** **4.40**, ***p** **<*** **0.001) pre-op vs. 1mpost-op t** **=** **5.72**, ***p** **<*** **0.001)**

ADLs, activities of daily living; QOL, quality of life; TETRAS, The Essential Tremor Rating Assessment Scale; QUEST, Quality of Life in Essential Tremor Questionnaire Linear mixed model included the measurement time-point (preoperatively [pre-op], 1-week post-operatively [1wpost-op], and 1-month postoperatively [1mpost-op]) as independent variables, age and sex as covariates, and participants as random effects. Bold font indicates significant correlations.

### 3.3. Motor symptoms

**Figure 2:**
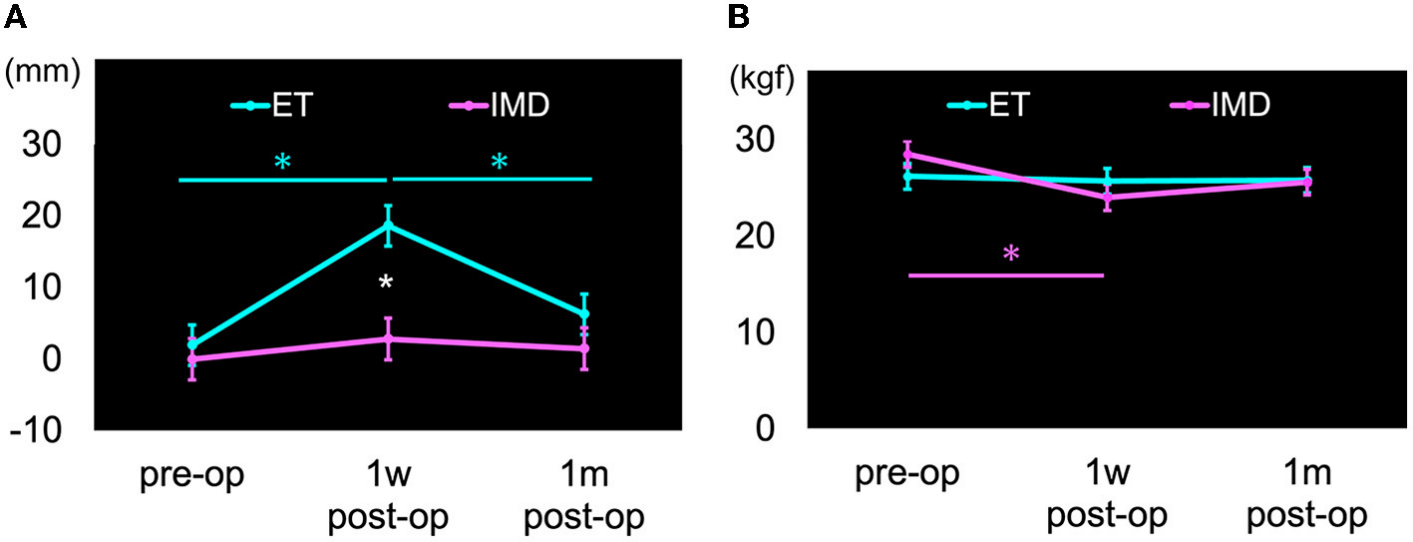
Motor symptoms with significant differences in time-group interaction. Cyan color: Essential tremor group (ET), Magenta color: Involuntary movement disorders group (IMD), Error bars: standard errors (SE), *p < 0.05. **(A)** Center of pressure horizontal displacement (COP-X). The direction of displacement to the treated side was calculated as a positive value. The ET group had significantly displaced COP-X to the treated side at 1wpost-op than at pre-op and significantly reduced displacement at 1mpost-op than at 1wpost-op, as shown in cyan color asterisks (*). The displacement in the ET group at 1wpost-op was significantly displaced compared to the IMD group, as shown by white color asterisk (*). **(B)** Strength grasp on the treated side. The grip strength on the treated side in the IMD group significantly decreased at 1wpost-op than at pre-op, as shown in magenta color asterisk (*).

For motor symptoms, a significant time-point × group interaction was found for an eccentric COP-X displacement to the treated side (F = 4.563, *p* = 0.015) and grip strength on the treated side (F = 4.990, *p* = 0.010) (Table 3). In the simple main effect results, the ET group showed a significantly greater eccentric COP-X displacement to the treated side at 1wpost-op than at pre-op (t = 4.871, *p* < 0.001) and significantly reduced displacement at 1mpost-op than at 1wpost-op (t = −3.601, *p* < 0.001), as shown in Figure 2A. In comparison, grip strength on the treated side in the IMD group showed a more significant decrease at 1wpost-op than at pre-op (t = −4.935, *p* < 0.001), as shown in Figure 2B.

**Table 3:** Motor and non-motor symptoms and physical performance in participants with ET and IMD, post-radiofrequency ablation.

	**Essential tremor (ET)**			**Involuntary movement disorders (IMD)**			**Linear mixed model**		
**Variables above: mean (SE) below: estimated marginal mean (SE)**	**Pre-op**	**1-week post-op**	**1-month post-op**	**pre-op**	**1-week post-op**	**1-month post-op**	**Interaction group** × **measurement time-point**	**Main effect of measurement time-point**	**Main effect of the group**
**Motor symptoms**									
COP RMA	87.5 (13.5) 83.3 (48.6)	175 (40.0) 170.6 (48.6)	114 (21.4) 109.4 (48.6)	140 (48.7) 145.5 (50.3)	179 (79.9) 184.8 (50.3)	131 (39.0) 136.6 (50.3)	F_(2, 56)_ = 0.3437 *p* = 0.711	F_(2, 56)_ = 2.6223 *p* = 0.082	F_(1, 26)_ = 0.2886 *p* = 0.596
COP LNG	267 (21.8) 253 (36.3)	327 (30.9) 313 (36.3)	253 (17.6) 239 (36.3)	281 (30.3) 300 (37.6)	304 (60.6) 324 (37.6)	261 (32.1) 280 (37.6)	F_(2, 56)_ = 0.384 *p* = 0.683	**F**_**(2, 56)**_ **=** **3.730** ***p*** **=** **0.030**	F_(1, 26)_ = 0.478 *p* = 0.495
COP-X	1.63 (2.55) 1.9487 (2.82)	18.3 (2.82) 18.6195 (2.82)	5.97 (2.39) 6.2965 (2.82)	0.44 (2.34)−0.0149 (2.89)	3.26 (1.96) 2.8051 (2.89)	1.92 (3.81) 1.4651 (2.89)	**F**_**(2, 56)**_ **=** **4.563** ***p*** **=** **0.015**	**F**_**(2, 56)**_ **=** **8.544** ***p** **<*** **0.001**	**F**_**(1, 26)**_ **=** **5.981** ***p*** **=** **0.022**
COP-Y	−7.49 (3.44) −7.637 (3.37)	−10.7 (2.75) −10.865 (3.37)	−5.93 (3.52) −6.080 (3.37)	−1.64 (2.66) −1.032 (3.47)	−7.16 (3.53) −6.552 (3.47)	−0.253 (3.45) 0.355 (3.47)	F_(2, 56)_ = 0.1123 *p* = 0.894	F_(2, 56)_ = 2.5476 *p* = 0.087	F_(1, 26)_ = 2.1780 *p* = 0.152
Grip strength on treated side	27.6 (2.13) 26.2 (1.32)	27.1 (2.51) 25.7 (1.32)	27.2 (2.19) 25.8 (1.32)	25.0 (2.72) 28.5 (1.34)	20.5 (2.18) 24.0 (1.34)	22.1 (2.63) 25.6 (1.34)	**F**_**(2, 56)**_ **=** **4.99023** ***p*** **=** **0.010**	**F**_**(2, 56)**_ **=** **7.72901** ***p*** **=** **0.001**	F_(1, 26)_ = 0.00646 p = 0.937
Grip strength on the non-treated side	25.5 (2.23) 24.3 (1.26)	25.2 (2.27) 24.0 (1.26)	26.0 (2.43) 24.9 (1.26)	22.8 (2.42) 26.1 (1.28)	20.9 (2.04) 24.2 (1.28)	23.1 (2.53) 26.4 (1.28)	F_(2, 56)_ = 0.8668 *p* = 0.426	F_(2, 56)_ = 2.7482 p = 0.073	F_(1, 25)_ = 044009 *p* = 0.513
**Non-motor symptoms**									
MMSE	28.5 (0.361) 28.6 (0.537)	28.2 (0.564) 28.4 (0.537)	29.3 (0.356) 29.5 (0.537)	27.6 (0.622) 27.4 (0.555)	27.6 (0.698) 27.4 (0.555)	28.9 (0.385) 28.7 (0.555)	F_(2, 56)_ = 0.150 *p* = 0.861	**F**_**(2, 56)**_ **=** **6.426** ***p*** **=** **0.003**	F_(1, 26)_ = 2.115 *p* = 0.158
Verbal fluency-category	12.2 (0.865) 12.4 (1.08)	12.9 (0.866) 13.0 (1.08)	13.2 (0.682) 13.3 (1.08)	17.6 (0.881) 17.1 (1.12)	16.0 (1.66) 15.6 (1.12)	14.8 (1.2) 14.3 (1.12)	**F**_**(2, 56)**_ **=** **4.720** ***p*** **=** **0.013**	F_(2, 56)_ = 1.116 *p* = 0.319	F_(1, 26)_ = 3.492 *p* = 0.073
Verbal fluency-phoneme	7.82 (0.693) 7.92 (0.952)	6.98 (0.683) 7.07 (0.952)	8.3 (0.871) 8.40 (0.952)	13.7 (1.03) 13.63 (0.984)	8.67 (1.08) 8.56 (0.984)	11.4 (0.952) 11.27 (0.984)	**F**_**(2, 56)**_ **=** **5.71776** ***p*** **=** **0.006**	**F**_**(2, 56)**_ **=** **11.24005** ***p** **<*** **0.001**	**F**_**(1, 26)**_ **=** **7.55014** ***p*** **=** **0.011**
**Physical performance**									
10MWT	5.26 (0.167) 5.29 (0.247)	5.88 (0.169) 5.91 (0.247)	5.67 (0.3) 5.71 (0.247)	4.85 (0.174) 4.75 (0.255)	5.77 (0.344) 5.68 (0.255)	5.2 (0.226) 5.10 (0.255)	F_(2, 56)_ = 0.6371 *p* = 0.533	**F**_**(2, 56)**_ **=** **9.7459** ***p** **<*** **0.001**	F_(1, 26)_ = 2.2021 *p* = 0.150
BBS	55.3 (0.371) 55.4 (0.586)	52.8 (0.719) 52.9 (0.586)	54.1 (0.524) 54.2 (0.586)	55.3 (0.347) 55.3 (0.605)	54.7 (0.836) 54.7 (0.605)	55.0 (0.468) 55.0 (0.605)	F_(2, 56)_ = 2.353 *p* = 0.104	**F**_**(2, 56)**_ **=** **6.466** ***p*** **=** **0.003**	F_(1, 26)_ = 1.330 *p* = 0.259
STEF on treated side	91.7 (1.61) 91.6 (1.59)	85.5 (1.7) 85.5 (1.59)	90.0 (1.72) 89.9 (1.59)	98.7 (0.599) 98.8 (1.65)	93.9 (1.66) 94.1 (1.65)	96.1 (1.39) 96.2 (1.65)	F_(2, 56)_ = 0.7679 *p* = 0.469	**F**_**(2, 56)**_ **=** **16.3252** ***p** **<*** **0.001**	**F**_**(1, 26)**_ **=** **12.1260** ***p*** **=** **0.002**
STEF on the non-treated side	91.4 (1.83) 90.9 (1.47)	88.6 (2.04) 88.1 (1.47)	90.6 (1.9) 90.0 (1.47)	98.9 (0.446) 99.4 (1.53)	98.3 (0.575) 98.8 (1.53)	98.1 (0.661) 98.6 (1.53)	F_(2, 56)_ = 1.8724 *p* = 0.163	**F**_**(2, 56)**_ **=** **3.1987 p** **=** **0.048**	**F**_**(1, 26)**_ **=** **19.5081** *p < * **0.001**

BBS, Berg Balance Scale; COP, center of pressure; ET, essential tremor with Vim-targeted-radiofrequency ablation; IMD, Involuntary movement disorders with non-Vim-targeted-radiofrequency ablation; LNG, length; MMSE, Mini-Mental State Examination; RMA, root mean square area; SE, standard error; STEF, simple test for evaluating hand function; 10MWT, 10-meter walk test. Linear mixed model included Group (ET and IMD) × Measurement time-point (preoperatively [pre-op], 1-week post-operatively [1wpost-op], and 1-month postoperatively [1mpost-op]) as independent variables, age and sex as covariates (with weight added as a covariate for grip strength), and participants as random effects. Bold font indicates significant correlations.

A main effect of time of measurement, but no interaction, was seen for COP-LNG. COP-LNG was more prolonged at 1wpost-op than at 1mpost-op (t = −2.658, *p* = 0.031). There was no statistically significant change from pre-op to 1wpost-op (t = 1.872, *p* = 0.199) or 1mpost-op (t = −0.787, *p* > 0.999) (Table 3).

### 3.4. Non-motor symptoms

Significant time-point × group interactions were seen among non-motor symptom outcomes for VF-p and VF-c. The VF-p score in the IMD group at 1wpost-op was lower than that at pre-op (t = −5.625, *p* < 0.001) and at 1mpost-op (t = 3.010, *p* = 0.004). The VF-c score did not change significantly in either group. The MMSE scores showed a main effect of the measurement time-point but no interaction. The MMSE score at 1mpost-op was significantly improved than that at pre-op (t = 2.887, *p* = 0.017) and at 1wpost-op (t = 3.284, *p* = 0.005) (Table 3).

### 3.5. Physical performance

The 10-MWT, STEF scores on the treated and non-treated sides and the BBS score did not show any statistically significant interaction or effects on the measurement time-point. The STEF scores on the treated side were significantly improved at 1mpost-op than that at 1wpost-op (t = 3.44, *p* = 0.003). While both STEF scores showed main effects on the group, STEF scores on the treated and non-treated sides in the ET group were significantly worse than those in the IMD group (F = 12.126, *p* = 0.002 and F = 19.5081, *p* < 0.001, respectively) (Table 3).

### 3.6. Correlation of motor and non-motor symptoms with physical performance decline

Correlation analyses were analyzed between the pre-op or 1wpostop motor or non-motor symptoms with changes due to RFA. The ET group had a correlation between COP-X and BBS (r = −0.661, *p* = 0.007). In the IMD group, grip strength on the treated side correlated with STEF on the treated side (r = 0.520, *p* = 0.047) and VF-c correlated with the 10-MWT (r = −0.696, *p* = 0.004) (Table 4).

**Table 4:** Correlations of the amount of change between preoperative and 1-week postoperative motor and non-motor symptoms with physical performance decline in ET and IMD.

**Values mean (SE)**	**STEF on treated side −6.19 (1.78)**	**STEF on non-treated side −2.78 (1.78)**	**10-MWT 0.623 (0.139)**	**BBS −2.42 (0.752)**
**(A) ET**				
COP X 16.7 (3.93)	r = 0.304 *p* = 0.271	r = −0.338 *p* = 0.217	r = 0.310 *p* = 0.261	**r** **=** **−0.661** ***p*** **=** **0.007**
**(B) IMD**				
**Values mean (SE)**	**STEF treated side** −**4.73 (1.42)**	**STEF non-treated side** −**0.533 (0.446)**	**10-MWT 0.927 (0.304)**	**BBS** −**0.600 (0.623)**
Grip strength on treated side−4.53 (1.31)	**r** **=** **0.520** ***p*** **=** **0.047**	r = 0.514 *p* = 0.050	r = −0.462 *p* = 0.083	r = 0.164 *p* = 0.560
Verbal fluency–category−1.51(1.18)	r = 0.318 *p* = 0.248	r = 0.184 *p* = 0.512	**r** **=** **−0.696** ***p*** **=** **0.004**	r = 0.437 *p* = 0.103
Verbal fluency–phenomenon –**5.07 (1.06)**	r = 0.070 *p* = 0.803	r = 0.175 *p* = 0.534	r = −0.270 *p* = 0.330	r = 0.118 *p* = 0.676

BBS, Berg Balance Scale; ET, Essential tremor with Vim-targeted-radiofrequency ablation; IMD, Involuntary movement disorders with non-Vim-targeted-radiofrequency ablation; SE, standard error; STEF, simple test for evaluating hand function; 10-MWT, 10-meter walk test. Pearson's product-moment correlation coefficients. Bold font indicates significant correlations.

## 4. Discussion

In this case–control study, we evaluated the symptoms of individuals with ET, treated with Vim-targeted RFA, during the perioperative period compared to individuals with IMD treated with RFA targeting non-Vim areas. We found that individuals with ET who underwent Vim-targeted RFA showed improvement in tremors, ADLs, and QOL. However, they showed a significant shift in COP-X toward the treated side (dominant side of the RFA target's hemisphere) 1 week after RFA compared to the preoperative period. To the best of our knowledge, this is the first study to elucidate functional decline after RFA using quantitative measures in patients with ET as compared to control participants. The COP-X displacement can help to assess symptoms other than tremors arising after RFA and can provide valuable information as a clinical indicator of response to rehabilitation methods and outcomes.

The Vim is innervated by the dentato-rubro-thalamic tract of the dentate nucleus of the cerebellum, which has neural connections to the primary motor cortex in the cerebrum, forming the cerebellar-thalamo-cortical network (CTC) (28). CTC network abnormalities in ET are primarily attributable to pathophysiology (29) and swelling surrounding the permanent brain lesion after RFA. However, the COP displacement to the tremor side cannot be fully explained based on the effect of the Vim-targeted RFA on the CTC network. The total trajectory length and rectangular area, which directly measured the amount of center-of-gravity motion, did not change significantly between the pre- and post-surgery period in this study.

We hypothesized that tremor symptom improvement after RFA occurred as a shift in balance retention and postural strategy. Pre-operatively, individuals with ET adjusted the center of gravity to the median direction by applying torque in the direction of the treated side to maintain balance against the presence of unbalanced tremor symptoms. Since postoperative measures to maintain posture reduced the symptoms of tremors, it would be necessary to readjust the torque to the tremor side. ET individuals were reported to have impaired motor learning, which caused a functional disturbance of olivo-cerebellar circuits in the pathogenesis of ET (30). Therefore, this readjustment takes time due to impaired motor learning, the torque toward the tremor direction may be excessive until the patient adapts, and the center-of-gravity may shift in the tremor direction.

In individuals with IMD who underwent non-Vim-targeted RFA, treated-side grip strength and phonic VF worsened 1 week after surgery. The targets of RFA were the Vo, GPi, or PTT, which are included in the basal ganglia–thalamocortical (BTC) network (29). The BTC network, linked to both the RFA targets used in IMD individuals and to the gray matter of the supplementary motor cortex, is associated with smooth motor coordination (31) and has also been shown to regulate grip strength (32). It is reasonable to conclude that RFA-induced changes in the BTC network cause a perioperative decline in grip strength in individuals with IMD. In this study, the IMD group consisted of dystonia patients with RFA targets other than Vim, and neither the dystonia site nor the RFA target was controlled. The postoperative changes of each dystonia site were consecutive. We consider that individuals with cervical dystonia might have differed from the other types (Supplementary Table 1). Further investigation of the relationship between the dystonia site and the RFA target is necessary to determine the cause of the decrease in grip strength after RFA surgery.

A systematic review of the neural basis of VF using functional magnetic resonance imaging (fMRI) suggested that phonic-related VF is more associated with the dorsal left inferior frontal gyrus than meaning-related VF (33). In comparison, the GPi, one of the RFA targets in IMD, is located in the cognitive loop, involved in the cortico-basal ganglia circuits. We speculated that the low VF-p score at 1w-postop in the IMD group was due to the inability to inhibit the habitual response of using words according to their meaning, due to the deterioration of the prefrontal cortical loop, similar to the VF-p results seen in Parkinson's disease patients who have undergone GPi-targeted DBS (34).

Physical disabilities may occur due to craniotomy, and they can also be interpreted as disabilities due to the different neural mechanisms associated with each targeted RFA site. The current results of the correlation analyses between the COP-X and BBS in the ET group, and between the treated-side grip strength and STEF in the IMD group, supports this interpretation. Perioperative physical disabilities were not found to be specific to the RFA target, and both groups had worse physical performance post-treatment than preoperatively. Therefore, we propose that rehabilitation for physical disabilities in the perioperative period should consider the impact of functional impairment on the target site. Future studies should include symptom progression, deep somatosensory function, and limb muscle strength to strengthen the findings of the present study.

The current study had three limitations for generalizing post-RFA symptoms in individuals with ET. First, we investigated patients with ET who underwent RFA performed by a surgeon at a single institution. Second, the current data included imputed missing values due to missing data points for some participants. Third, to make the relationship between post-RFA symptoms and physical performance decline, a more robust finding and a large sample size are needed to allow multivariate regression analysis in a future study.

## 5. Conclusion

We compared symptoms after RFA treatment between individuals in ET, among whom the Vim was targeted, and individuals with IMD, among whom other brain regions were targeted, in a longitudinal case–control study, with measurement performed at 1-week and at 1-month post-RFA. We showed that individuals in the ET group showed improvement in tremors, ADL, and QOL at 1-month post-RFA. However, the COP showed horizontal displacement to the treated side at 1-week post-RFA. This COP displacement was associated with balance dysfunction related to postural stability post-RFA. Our theory is that the shift in the center of pressure toward the treated side may be caused by a delay in adapting to the long-term differences in tremor symptoms, which improve after RFA. We propose that the horizontal displacement of the center of pressure can be used to evaluate non-tremor symptoms that occur after RFA, providing important information as an indicator of the effectiveness of rehabilitation methods and their results.

## Data availability statement

The raw data supporting the conclusions of this article will be made available by the authors, without undue reservation.

## Ethics statement

The studies involving human participants were reviewed and approved by the Ethical Review Committee of the Tokyo University of Health Sciences in Tokyo University of Health Sciences (TPU-20-004) and the Sanai Hospital Ethical Review Committee in Sanai Hospital (21-s001). The patients/participants provided their written informed consent to participate in this study.

## Author contributions

The study was designed by AS and TI with contributions from TH and MK. Patient identification and surgery were carried out by TT. Subjects recruitment and data collection were carried out by KK and TA. Data analysis was carried out by AS and TI. The article was primarily written by AS and TI, and reviewed by all the authors. All authors contributed to the article and approved the submitted version.
